# Flexible intervention workflow for MRI guided robotic prostate seed brachytherapy

**DOI:** 10.1002/acm2.70747

**Published:** 2026-08-16

**Authors:** Philipp Aumueller, Mirjam L. Dannert, Martin Polednik, Frank A. Giordano, Jens Fleckenstein, Sven Clausen

**Affiliations:** ^1^ Department of Radiation Oncology University Medical Centre Mannheim, University Heidelberg Mannheim Germany; ^2^ Research Campus M^2^OLIE University Medical Centre Mannheim, University Heidelberg Mannheim Germany; ^3^ DKFZ‐Hector Cancer Institute at the University Medical Center Mannheim Mannheim Germany; ^4^ Mannheim Institute for Intelligent Systems in Medicine (MIISM) Mannheim Germany

**Keywords:** brachytherapy, focal therapy, MRI, robotic guidance, seed placement

## Abstract

**Background:**

Low‐dose‐rate prostate seed brachytherapy (BT) is an established guideline‐conformal treatment option for low‐risk prostate cancer. Precise seed placement is essential for achieving adequate dose coverage while minimizing exposure of surrounding healthy tissue. MRI guidance offers superior soft‐tissue contrast for target visualization, but technical challenges remain for accurate transrectal seed implantation under in‐bore conditions.

**Purpose:**

The Remote Control Manipulator (RCM) is an MRI‐compatible robotic guidance system originally designed for in‐bore transrectal targeted prostate biopsies. This work investigates its feasibility for MRI‐guided transrectal targeted focal prostate seed BT. We demonstrate the intervention workflow and quantify seed placement accuracy in a preclinical phantom experiment.

**Methods:**

The proposed intervention workflow defines the setup steps, interaction of the treatment planning system (TPS) with the robot's navigation software, MR‐imaging as well as seed‐ and needle‐guide registration. We performed and analyzed the total workflow in an anthropomorphic pelvis phantom. Seed positions corresponding to the initial treatment plan, the expected positions after needle‐guide alignment, and the final released seed positions were assessed in detail. Their geometric displacement was decomposed into contributions from robotic alignment, seed release, and registration uncertainty. Dosimetric consequences were evaluated using the target's V_100%_ dose coverage.

**Results:**

Robotic alignment produced a mean expected seed displacement of 0.6 ± 0.2 mm. Total deviation from planned to released seed positions was 2.6 ± 0.9 mm, with 0.7 ± 0.4 mm along‐trajectory displacement. MRI‐only seed registration differed from CT‐verified reconstruction by 2.8 ± 1.4 mm. After two released needles, V_100%_ declined from 100% to 92.9% using MRI‐only data and to 85.7% using CT‐verified seed positions.

**Conclusions:**

The workflow was successfully executed and demonstrated sub‐millimeter robotic targeting precision. Seed displacement during release and limited MRI‐only seed localization remain dominant sources of error impacting dose coverage. Active needle guidance during imaging and improved MRI‐only seed reconstruction could enable monitoring the live dose distribution and adaptive trajectory correction.

## 1 INTRODUCTION

Permanent ^125^I seed brachytherapy (BT) is a well‐established, minimally invasive treatment option for localized prostate cancer^1^ offering favorable tumor control and long term quality of life.^2^, ^3^ Standard‐of‐care implantation is typically performed under transrectal ultrasound (TRUS) guidance using a fixed template grid.^4^ While this technique is clinically validated, it has intrinsic limitations in anatomical visualization and needle placement precision due to limited soft‐tissue contrast and long transperineal needle paths (> 10 cm).

Magnetic resonance imaging (MRI) provides excellent soft‐tissue contrast and accurate intraprostatic lesion visualization, particularly suited for focal dose escalation strategies.^5^ Combining this imaging advantage with robotic needle steering has the potential to improve targeting accuracy,^6^, ^7^ reduce needle displacement, and enable focal‐lesion‐adapted implantation. In contrast to the standard TRUS‐guided approach, a transrectal MRI‐guided approach offers shorter needle trajectories (∼2 cm), potentially reducing needle deflection.^8^, ^9^, ^10^

The Remote Control Manipulator (RCM, Soteria Medical, Arnhem, the Netherlands) is an MRI‐compatible FDA approved robotic guidance system, designed for in‐bore transrectal prostate biopsies.^11^, ^12^ It allows fan‐shaped needle trajectories around a rotation point that is kept fixed during alignment. The needle guide contains a water filled reservoir around the stich canal allowing visualization and automatic registration on MRI. This capability offers the opportunity to replace the conventional perineal template with a flexible, image‐guided needle steering approach.

The aim of this study is to evaluate the feasibility of MRI‐guided robotic seed implantation using the RCM and to quantify the accuracy of each step in the workflow: The robotic needle‐guide targeting, the needle insertion and seed release, MRI‐only‐ and CT‐verified seed registration. The resulting dosimetric consequences on the V_100%_ are monitored. An experimental seed implantation is conducted, using an anthropomorphic pelvis phantom. The seed placement accuracy (SPA) and dose coverage is assessed in a controlled pre‐clinical setting.

## 2 MATERIALS AND METHODS

### 2.1 RCM path and treatment planning

**FIGURE 1:**
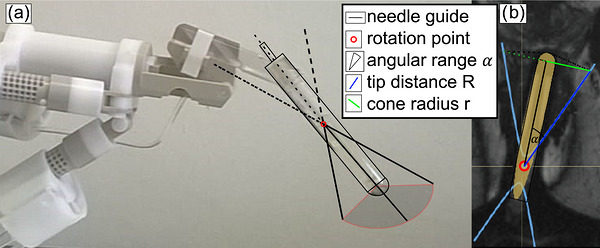
(a) RCM needle guide with a schematic illustration of its fan‐shaped motion around the rotation point. (b) Exemplary MRI view of the registered needle guide (marked yellow) with the still selectable rotation point, the corresponding motion range (light blue lines) and the cone radius (green line).

The RCM enables rotational adjustment of the needle guide around a defined rotation point, generating fan‐shaped needle trajectories (Figure 1). The rotation point is a user‐defined, adjustable coordinate along the needle guide and selected after needle guide registration in MRI. The distance between the needle‐guide tip and the rotation point determines the available range of motion of the needle guide. This motion range forms a conical surface along which the needle‐guide tip can move. As illustrated in Figure 1, the cone radius r is determined by the distance R between the rotation point and the needle‐guide tip and the maximum angular range of motion α by r=R∗sin(α). The maximum angular range was determined to be 25.75°, corresponding to a maximum cone radius of approximately 4.6 cm for R = 10.5 cm.

An in‐house developed treatment planning system (TPS) was adapted to incorporate the rotation‐point coordinate as the common geometric intersection of the fan‐shaped needle trajectories. Each trajectory was defined by the rotation point and the corresponding needle‐tip coordinate.^13^

Automatic inverse treatment planning was performed on alpha‐shape objects reconstructed from DICOM RT‐struct contours using the MATLAB function *alphaShape*.^14^ First, a greedy optimization approach is applied. Subsequent needle candidates are selected while satisfying dynamic dose‐range criteria [0.1 $D_{pr}$, $1.5\;D_{pr}+3N$], based on the prescription dose $D_{pr}$​ and the number of seeds N of the current needle candidate. The optimization process terminates when $V_{100\%}>97\%$ or $V_{150\%}>80\%$ is reached.

The resulting initial plan is subsequently refined using simulated annealing. Needle‐tip coordinates are randomly perturbed and optimized using the MATLAB *simulannealbnd* function.^15^ For each candidate configuration, the objective function evaluates the dose distribution and rewards target coverage while penalizing high dose deposition in normal tissue and organs at risk (OARs). Finally, all needle trajectories are checked for potential intersections with the urethra.

Following optimization, seeds located outside the focal clinical target volume (F‐CTV) are removed if their contribution to the dose objectives is negligible. These optimization steps are iteratively repeated as long as V_100_% > 96%, V_150_%> 79% holds and three percent of the prostate receive more than 115% of the prescription dose.

The TPS is based on the TG‐43 dose‐calculation formalism and previously demonstrated good agreement to our commercial treatment planning system (Oncentra Prostate, Nucletron‐v4.0) (OCP).^13^, ^16^ In contrast to OCP, which is based on template‐guided implantation, the proposed TPS is not restricted to parallel needle trajectories and can exploit the non‐parallel fan‐shaped needle geometry enabled by the robotic guidance system.

### 2.2 Robotic brachytherapy workflow

**FIGURE 2:**
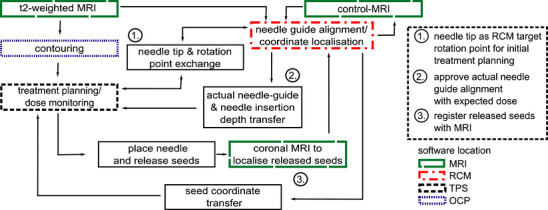
Workflow of the experimental procedure, starting in the upper left corner after setup preparation. The boxes indicate the software environments, namely magnetic resonance imaging (MRI), the Remote Controlled Manipulator (RCM), the treatment planning system (TPS) and Oncentra Prostate (OCP). The circled numbers (1.‐ 3.) denote the sequential data exchange between the systems.

**FIGURE 3:**
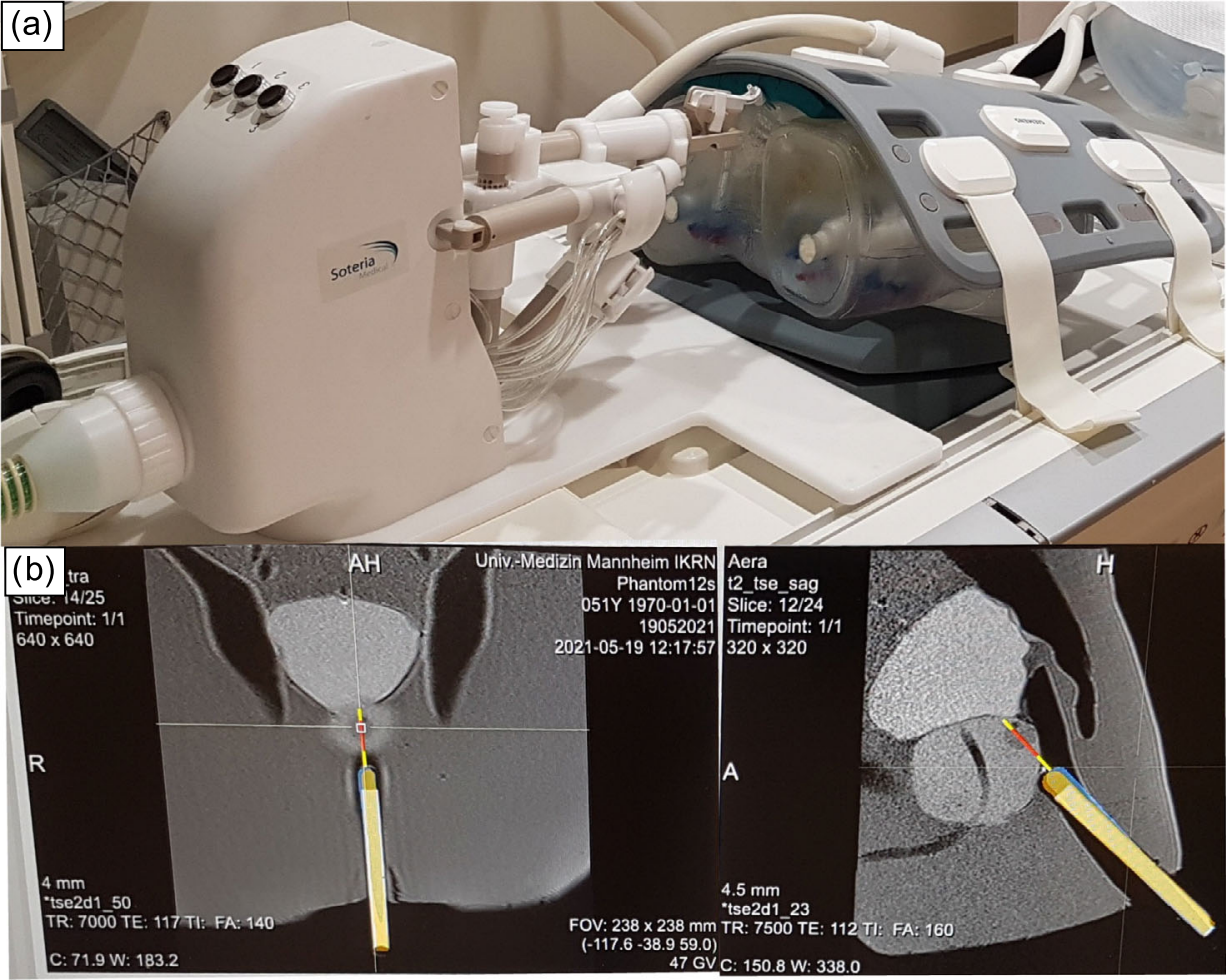
Overview of the experimental setup. (a) Implantation setup showing the RCM with the docked needle guide and the pelvic phantom positioned prone on the MRI table, using the standard body coil. (b) Transverse and sagittal MRI images of the phantom, showing the registered needle guide (yellow) and the planned needle guide position (blue).

The configuration of the phantom replicates the clinically established prone position used for in‐bore biopsies using the RCM. The workflow (Figure 2) begins with MRI acquisition using slice orientations aligned with the needle‐guide axis in both transversal and sagittal planes (Figure 3). These datasets are imported into the RCM software to verify robotic alignment and to define the rotation point along the needle guide considering CTV size and required cone radius as well as limitations due to rectum geometry.

The MRI data are subsequently transferred to OCP, where the prostate, focal target volume, needle guide, and organs at risk, such as urethra, bladder and rectum, are contoured.

The resulting DICOM RT‐struct file is imported into the TPS. A treatment plan is computed, and needles are preloaded with the respective number of seeds prior to implantation in the MRI control room.

During treatment, the first planned needle is transferred to the RCM application software. Since the rotation point is predefined, only the needle tip must be manually identified as target by localizing it in MRI. The needle guide is prompted to the desired location and verified using MRI. The needle‐guide tip and rotation‐point coordinates are extracted from these images to compute the expected needle alignment. Based on this alignment and the measured insertion depth from needle‐guide tip to target, expected seed positions and the resulting dose distribution are calculated. Fine adjustment of the needle‐guide position is performed if necessary.

To avoid magnetic interaction during insertion, the couch is retracted from the bore. The needle is advanced through the guide until the needle tip coincides with the needle‐guide tip. The needle is then inserted to the measured depth, and seeds are released. The couch is returned back to the previous in‐bore position, and a post implant MRI scan along the needle trajectory is acquired to manually register the released seeds. The procedure is repeated iteratively for all needles.

### 2.3 Phantom experiment

A recently developed anthropomorphic pelvis phantom^17^ designed for MRI‐guided prostate interventions was used. The phantom was positioned prone in a 1.5T MRI scanner (Magnetom Aera, Siemens Healthineers, Erlangen, Germany) with standard body coil configuration (Figure 3). High‐resolution T2‐weighted images (1 mm slice thickness, ∼45° slice angle according to the needle‐guide axis) were acquired for setup alignment. The initial planning position was determined by targeting the center of an easy reachable lesion. It was selected as focal clinical target volume (F‐CTV, 0.9 cc), which was expanded by 1 mm isotropic margin to define the focal planning target volume (F‐PTV, 1.3cc). This size is comparable to the smaller ones used by Brun et al.^18^ The rotation point was positioned centrally along the needle guide and within the rectum to minimize the angular displacement of both the needle guide tip and shaft relative to the initial trajectory, thereby reducing the risk of collision with the rigid rectal wall of the phantom. The resulting cone height was about 6 cm, corresponding to an expected maximum cone radius of 2.6 cm. Needle‐guide alignment was monitored using MRI, enabling iterative verification and correction prior to needle insertion. The RT struct was manually transferred to the TPS. A prescription dose of 160 Gy was assigned to cover the F‐PTV. The planning objective was V_100%_ close to 100%. The resulting inverse treatment plan comprised five needles with seven seeds in total. Commercially available MRI‐visible spacers (Sirius, C4 Imaging LLC, Texas, USA) were used to enable distinguishing between spacers and seeds. As a feasibility study, the robotic workflow was intentionally evaluated using two representative needles containing a total of four seeds rather than the complete treatment plan. This approach was chosen to demonstrate the technical implementation and workflow of robotic needle guidance and seed placement under MRI guidance while limiting experimental complexity. The selected needles were considered representative for assessing the accuracy and feasibility of the proposed system. All procedures were performed by a medical physicist experienced in phantom‐based prostate BT simulations but without prior clinical needle‐insertion experience.

### 2.4 Accuracy evaluation

**FIGURE 4:**
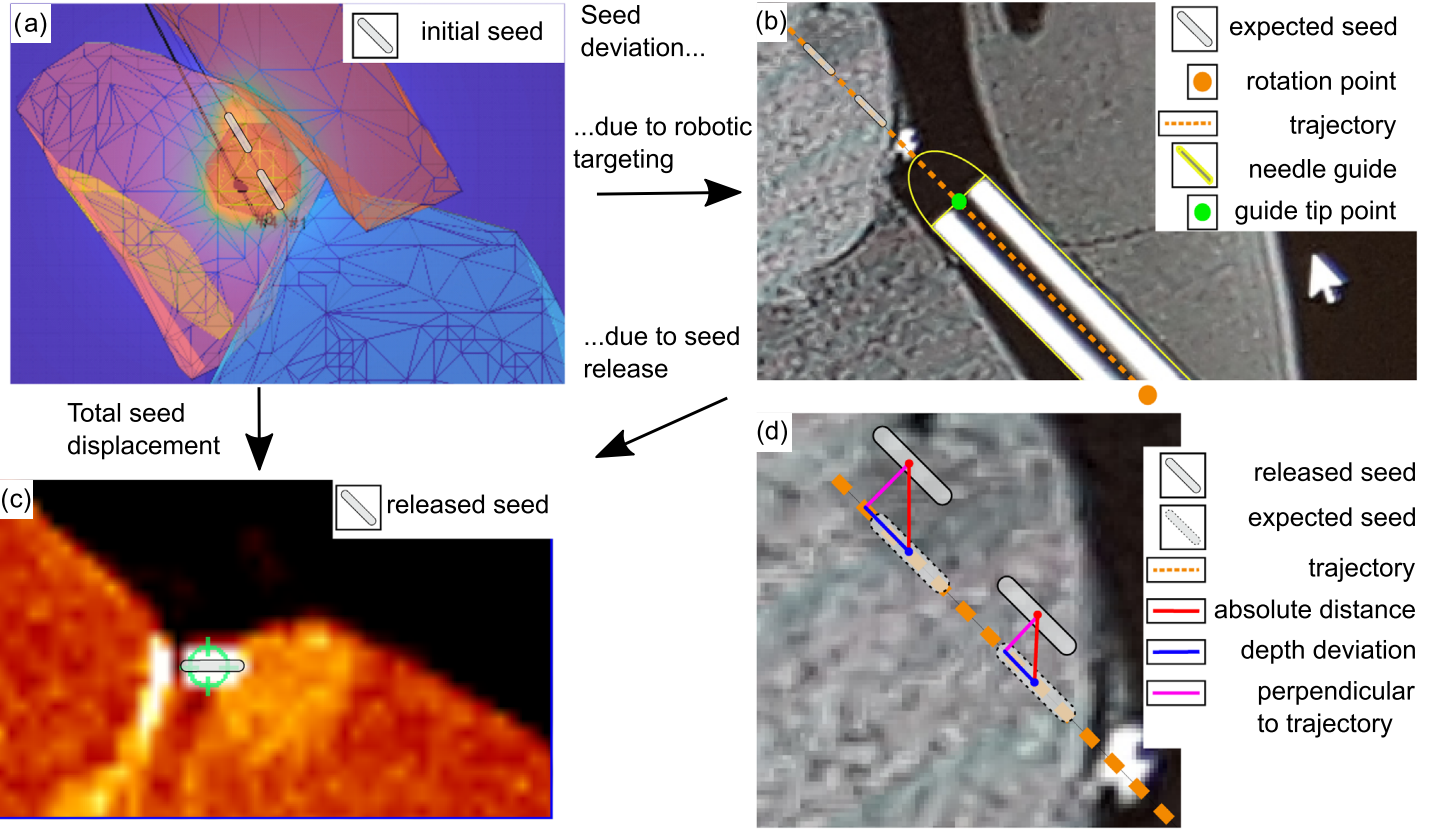
Seed states and corresponding deviation metrics. (a) Initial seed positions as defined in the treatment plan. (b) Expected seed positions after needle guide alignment. (c) Released seed visualized as positive contrast in CT imaging. (d) Illustration of depth deviation and absolute distance, using the example of expected versus released seeds positions.

Seed coordinates were determined at different stages (Figure 4). The initial seeds contain the planned positions from the treatment plan. Expected seeds contain the predicted positions based on the aligned needle‐guide position and insertion depth. Released seeds contain the observed positions in MRI or CT after implantation.

Seed deviation due to robotic targeting was defined as the absolute distance between initial and expected seed coordinates. Rotation point displacement was quantified for each needle.

Axial depth deviation along the needle trajectory was computed as the projection of the displacement vector onto the needle axis. The direction was recorded relative to the insertion direction with positive values for displacements into the phantom and vice versa.

For verification of MRI‐based seed registration, a CT scan was acquired post‐implantation. Rigid CT‐to‐MRI registration was performed and CT‐derived seed positions were transformed into the MRI coordinate system. The seed deviation due to registration error was defined as the absolute difference between MRI‐registered and CT‐registered seed positions.

The total seed displacement was defined as the absolute difference between initial and released seed positions. The seed deviation due to seed release was defined as the difference between expected and released positions.

V_100%_ coverage was computed for both MRI‐only and CT‐verified seed reconstructions.

## 3 RESULTS

### 3.1 Positioning accuracy

Table 1 summarizes spatial seed deviations and Table 2 the corresponding axial depth deviations. The seed deviation due to robotic targeting was 0.6 ± 0.2 mm (mean ±SD), confirming sub‐millimeter robotic alignment accuracy. The corresponding axial depth deviation was 0.4±0.1 mm in the forward insertion direction.

**TABLE 1:** Spatial deviations between the different seed states defined in this study (Δ). The needle number (#N) and seed number (#S) identify each implanted seed, with seed #1 located closest to the needle tip. Initial seeds (IN.) denote the planned seed positions derived from the treatment plan. Expected seeds (EXP.) represent the predicted seed positions calculated after final needle‐guide alignment and approval. Released seeds correspond to the observed post‐implant seed positions. The first column reports the seed deviation attributable to robotic needle‐guide targeting, defined as the distance between initial and expected seed positions (IN.–EXP.). The second and third columns report the total seed deviation between initial and released seed positions based on MRI‐only seed localization (MRI) and CT‐verified seed localization registered to MRI space (CT), respectively. The fourth and fifth columns report the seed deviation attributable to seed release, defined as the distance between expected and released seed positions. The final column reports the seed deviation attributable to image‐registration uncertainty, defined as the difference between MRI‐ and CT‐based released seed positions.

#N	#S	Δ(IN.–EXP.) (mm)	Δ(IN.–MRI) (mm)	Δ(IN.–CT) (mm)	Δ(EXP.–MRI) (mm)	Δ(EXP.–CT) (mm)	Δ(MRI–CT) (mm)
1	1	0.4	3.7	3.2	3.5	3.1	1.2
1	2	0.4	3.1	2.5	3.1	2.7	1.9
2	1	0.7	4.1	3.6	4.0	4.0	3.1
2	2	0.9	5.6	1.3	5.6	0.6	5.0
		0.6±0.2	4.1±0.9	2.6±0.9	4.1±0.9	2.6±1.2	2.8±1.4

**TABLE 2:** Axial needle depth deviations between the different seed states defined in this study (Δ). Positive values indicate displacement of the released seed position along the needle insertion direction into the phantom. The needle number (#N) and seed number (#S) identify each implanted seed, with seed #1 located closest to the needle tip. Initial seeds (IN.) denote the planned seed positions derived from the treatment plan, expected seeds (EXP.) the predicted seed positions calculated after final needle‐guide alignment, and released seeds the observed post‐implant seed positions. The first column reports the axial depth deviation attributable to robotic needle‐guide targeting, defined as the depth difference between initial and expected seed positions (IN.–EXP.). The second and third columns report the axial depth deviation between initial and released seed positions based on MRI‐only seed localization (MRI) and CT‐verified seed localization registered to MRI space (CT), respectively. The final two columns report the axial depth deviation between expected and released seed positions, representing the depth deviation attributable to seed release.

#N	#S	Δ(IN.–EXP.) (mm)	Δ(IN.–MRI) (mm)	Δ(IN.–CT) (mm)	Δ(EXP. –MRI) (mm)	Δ(EXP. –CT) (mm)
1	1	+0.3	−1.0	+1.0	−1.2	+0.7
1	2	+0.3	+0.9	0.0	+0.7	−0.3
2	1	+0.4	+3.4	+0.7	+3.1	+0.4
2	2	+0.4	+5.3	+1.1	+4.9	+0.6
		0.4±0.1	2.7±2.4	0.7±0.4	1.9±2.3	0.4±0.4

The total seed displacement in the CT‐verified data set were 2.6±0.9 mm. The corresponding seed deviation due to seed release was 2.6±1.2 mm, indicating that seed release contributed the dominant component of displacement. The respective axial depth deviations were 0.7±0.4 mm (total) and 0.4±0.4 mm due to seed release.

Insertion depths from needle‐guide tip to the target were 13.9 mm for the first needle and 19.7 mm for the second.

The rotation point shifted between alignment steps, leading to total deviations of 2.4 mm for the first needle and 3.3 mm for the second needle. Given the approximately 8 cm seed‐rotation‐point distance, these deviations contributed minimally to local seed displacement.

### 3.2 Detection accuracy

The seed deviation due to registration error was 2.8±1.4 mm. For the first needle, the deviations were 1.2 mm and 1.9 mm. For the second needle, one exhibited a deviation of 3.1 mm, while the second was misregistered in MRI by 5.0 mm.

Seed registration improved when MRI slices were orthogonal to the stitch canal, as each seed's longitudinal axis was then perpendicular to the imaging plane. For the first needle, preliminary non‐orthogonal registration yielded deviations of 2.0 mm and 12.5 mm, which were reduced to 1.2 mm and 1.9 mm after re‐evaluation using coronal views. The second needle was registered directly with coronal slices.

### 3.3 Dosimetric evaluation

The initial treatment plan achieved V_100% _= 100%. Expected seed positions (post needle‐guide alignment) did not alter this value. After implantation of the first needle, MRI‐only reconstruction yielded V_100% _= 92.9%, which remained stable after the second needle. However, a change of the geometric dose distribution was visible.

**FIGURE 5:**
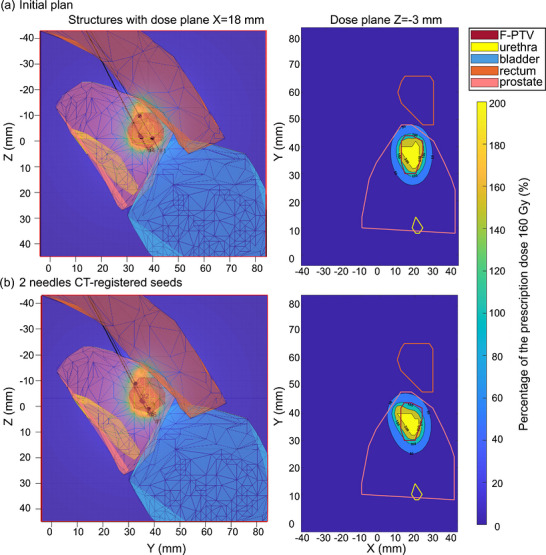
Contoured anatomical structures with corresponding dose distributions. The left panel shows the Y‐Z dose plane with X = 18 mm, while the right panel shows the contour plot of the X‐Y plane with Z = ‐3 mm. (a) Initial treatment plan. (b) Plan including two CT‐reconstructed needles and the remaining initial needles. The deviation of needle #1 is visible on the left panel, resulting in a shifted dose distribution on the right panel.

Using CT‐verified seed positions, V_100%_ declined to 91.1% after the first and 85.7% after completion of the second needle. In Figure 5 the dose distributions of the initial treatment plan for one sagittal and one transversal dose plane are compared against the one from the CT based evaluation after the release of the second needle's seeds. On the sagittal Y–Z dose plane (left panels), the first needle is predominantly displaced in the negative y‐direction, with only a minor deviation in the z‐direction. This displacement results in reduced dosimetric conformity toward the negative x‐direction on the transverse X–Y plane (right panels). The latter is consistent with the Δ(IN–CT) deviation of the first needle reported in Table 1. Furthermore, the small depth deviation reported in Table 2 is visually confirmed.

## 4 DISCUSSION

### 4.1 Positioning accuracy

The experiment demonstrated that the RCM provides highly accurate needle‐guide alignment with sub millimeter precision. The systematic rotation‐point drift of ∼2–3 mm had minimal effect on seed placement due to geometric scaling, but may become more relevant for extended seed strands in clinical settings.

Depth deviation analysis demonstrates that expected seed positions calculated after final alignment more closely approximate the true needle depth than planned positions, supporting the use of real‐time geometric adjustment. Manual seed release rather than robotic targeting was the dominant contributor to displacement. In total the seed placement accuracy was higher than state of the art seed‐BT which is assumed to be 3–6 mm.^19^

### 4.2 Mechanical effects during seed implantation

Ballistic gelatin produced substantial frictional resistance on the needle, which complicated precise seed releases. Slight clearance between the needle and the guide also leaded to lateral deflection.

The beveled needle tip, which is used to steer the needle during TRUS imaging, induced needle displacement as a result of needle bending. The needles were oriented such that the bevel tip pointed toward the initial needle tip coordinate, causing the needle bending to be directed toward the initial seed position. Consequently, the first seed of the second needle exhibited a reduced displacement of 3.6 mm compared to the expected displacement of 4.1 mm. However, the use of a symmetrically shaped needle tip may reduce needle bending and thereby improve targeting accuracy. This is especially useful when steering the needle without image guidance.

### 4.3 Detection accuracy

The observed seed deviation due to registration error of 2.8 mm was comparable to seed‐release displacement, demonstrating that manual MRI‐only localization is not sufficiently accurate for intra‐operative dose validation. Susceptibility artifacts due to seed material were the principal source of misregistration.^20^ Quantitative susceptibility mapping (QSM)‐based automatic seed localization^21^ or the use of MRI visible seed spacers^22^ may improve localization accuracy to better monitor the dose distribution during treatment.

At present, CT‐based registration remains the gold standard but is limited to post‐implant verification. In this study, the C4 spacers exhibited the same signal intensity as the phantom material and were therefore not clearly distinguishable. However, they did not produce signal voids that could have complicated seed detection.

### 4.4 Dose evaluation

The reduction of V_100%_ to 85.7% in CT‐verified reconstruction indicates that focal BT, using a small number of seeds, is highly sensitive to individual seed placement errors. Unlike whole‐gland BT, focal implantation lacks dose averaging.^23^ Consequently, continuous monitoring of the dose distribution is essential to ensure adequate dose coverage. Re‐optimization of the remaining needle positions and adding further seeds to underdosed regions are necessary options to restore target coverage, given the post implantation constraint of V_100% _> 95%.^24^ Furthermore, enlarging the F‐PTV could increase the number of implanted seeds and thereby enhance dose averaging. Reducing the source strength while raising the number of seeds on focal target volumes is another way to enhance dose averaging.^18^, ^25^ However, this might result in a reduced BED due to a lower initial dose rate^26^ as well as an increased inter‐seed attenuation effect^27^ due to the shorter inter‐seed distances.

### 4.5 Limitations and potentials of this study

A limitation of this study is that the robotic procedure was performed for only two of the five planned needles and four of the seven planned seeds. Consequently, a complete end‐to‐end validation of the full implantation procedure and a comprehensive statistical analysis of all seed placement errors were not performed. Therefore the post implantation dosimetry could not be investigated. Future work will focus on adaptive treatment planning strategies in which the actual dose after each needle placement is used to re‐optimize subsequent needle trajectories and seed positions.

The current workflow is constrained by the fixed‐rotation‐point geometry of the RCM, which does not support dynamic repositioning of the rotation point during alignment or insertion. This limitation reduces the feasibility to realign the needle guide especially for needles with long seed strands.

The phantom used does not fully replicate human tissue elasticity, deformation or motion. It could not be positioned as optimally as a patient during in‐bore biopsies, due to the absence of hip joints that would facilitate the reachability of certain targets. Additionally, only focal BT with short pathways was tested, and feasibility for whole prostate coverage remains unverified.

In our department the maximum prostate CTV treated with TRUS guided seed‐BT was limited to 60 mL, as the insertion of parallel needles can be obstructed by the hip bones in larger prostates. Assuming a larger spherical prostate volume of 70 mL, the radius from center to gland boundary would be approximately 2.6 cm. To accommodate the corresponding insertion cone, the distance R from the rotation point to the needle guide tip would need to be at least 6 cm (see Section 2.1). Given the maximum angular range, a spherical target volume of approximately 400 mL could theoretically be reached with R= 10.5 cm. From a robotic motion perspective, the treatment of substantially larger prostates therefore appears feasible and could potentially extend the range of treatable prostate volumes beyond that achievable with conventional transperineal seed brachytherapy.

Technical improvements could include symmetric MRI‐safe needles, optimized MRI sequence protocols, or automated seed‐registration algorithms. With upcoming low‐field strength MRI scanners the standard implantation needles could become MRI safe such that they could be used inside of the bore.^28^ Integration of real‐time seed localization into the TPS could allow adaptive dose compensation during implantation. The use of stranded seeds instead of individual seeds and spacers may prevent shifts within the seed chain during seed release and thereby reduce the overall seed displacement error.

## 5 CONCLUSION

This study establishes the technical feasibility of MRI‐guided robotic seed BT using a transrectal needle‐guide system with submillimeter alignment accuracy. While robotic targeting demonstrates high precision, seed displacement during release and MRI‐based localization error limit overall dosimetric conformity. Accurate localization of seed positions during the procedure, combined with adaptive trajectory adjustment, represents a necessary foundation for clinical translation. Technical refinements in needle design and accurate MRI seed registration could enable real‐time dose monitoring and adaptive planning, supporting precise focal dose delivery.

## AUTHOR CONTRIBUTIONS

**Philipp Aumueller**: Conceptualization; experimental part; programming; data analysis; writing of original draft. **Mirjam L. Dannert**: Experimental part; programming. **Martin Polednik**: Review and editing. **Sven Clausen**: Conceptualization; review and editing; supervision. **Frank A. Giordano**: Conceptualization; review and editing. **Jens Fleckenstein**: Review and editing.

## CONFLICT OF INTEREST STATEMENT

The authors declare that they have no known competing financial interests or personal relationships that could have appeared to influence the work reported in this paper.

## Data Availability

All relevant data are included in this article. Additional data are available from the corresponding author upon reasonable request.
